# The Thermal, Electrical and Thermoelectric Properties of Graphene Nanomaterials

**DOI:** 10.3390/nano9020218

**Published:** 2019-02-06

**Authors:** Jingang Wang, Xijiao Mu, Mengtao Sun

**Affiliations:** 1 Computational Center for Property and Modification on Nanomaterials, College of Sciences, Liaoning Shihua University, Fushun 113001, China; jingang_wang@lnpu.edu.cn; 2 Center for Green Innovation, Beijing Key Laboratory for Magneto-Photoelectrical Composite and Interface Science, School of Mathematics and Physics, University of Science and Technology Beijing, Beijing 100083, China; shumuxijiao@163.com

**Keywords:** graphene, electrical, thermal, thermoelectric, applications

## Abstract

Graphene, as a typical two-dimensional nanometer material, has shown its unique application potential in electrical characteristics, thermal properties, and thermoelectric properties by virtue of its novel electronic structure. The field of traditional material modification mainly changes or enhances certain properties of materials by mixing a variety of materials (to form a heterostructure) and doping. For graphene as well, this paper specifically discusses the use of traditional modification methods to improve graphene’s electrical and thermoelectrical properties. More deeply, since graphene is an atomic-level thin film material, its shape and edge conformation (zigzag boundary and armchair boundary) have a great impact on performance. Therefore, this paper reviews the graphene modification field in recent years. Through the change in the shape of graphene, the change in the boundary structure configuration, the doping of other atoms, and the formation of a heterostructure, the electrical, thermal, and thermoelectric properties of graphene change, resulting in broader applications in more fields. Through studies of graphene’s electrical, thermal, and thermoelectric properties in recent years, progress has been made not only in experimental testing, but also in theoretical calculation. These aspects of graphene are reviewed in this paper.

## 1. Introduction of Graphene

**Figure 1:**
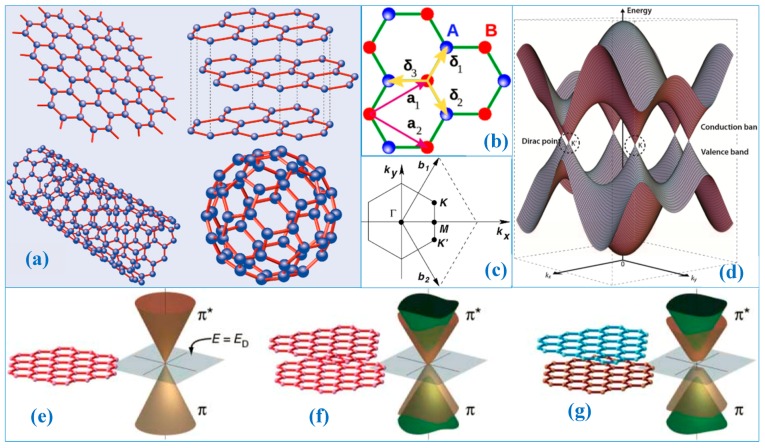
(**a**) Carbon-based nanomaterials [2]. Copyright 2008, Springer. (**b**) The planar crystal structure of graphene: $a_{1}$ and $a_{2}$ are the lattice vectors of the unit cell; δ_1_, δ_2_, and δ_3_ are vectors mutually adjacent to each other [3]. (**c**) The Brillouin zone (BZ) corresponding to the graphene lattice (b_1_ and b_2_ are the reciprocal space bases; K, M, K’, etc. represent k-space high symmetry points. The coordinate axis is the k-space basis vector.) [3]. Copyright 2009, American Physical Society. (**d**) Energy band diagram of graphene’s hexagonal lattice [4]. Copyright 2009, Springer. Electronic structure of a single layer (**e**), symmetric double layer (**f**), and asymmetric double layer (**g**) of graphene. The energy bands depend only on in-plane momentum because the electrons are restricted to motion in a two-dimensional plane. The Dirac crossing points (marked by a grey cross and square) are at energy ***E***_D_ [5]. Copyright 2006, Science.

As an ideal two-dimensional (2D) material, graphene has become a popular topic of scientific research due to its distinctive physical and chemical properties since it was first proposed in 2004 [1,2,3]. Monolayer graphene has a hexagonal honeycomb structure comprised of the thinnest 2D crystal; graphene film is only one atom thick [2]. The *sp^2^* hybridization of C atoms also gives graphene its novel properties. As the basic unit of C-based materials, graphene can form zero-dimensional fullerenes, curl into one-dimensional C nanotubes, and form graphite by means of AA stacking or AB stacking; see Figure 1a [2].

Graphene is a transparent conductor that can be used to replace the current liquid crystal display materials [5]. It is one of the smallest known inorganic nanomaterials, is very strong and hard, and is 100 times stronger than the best steel in the world [6]. The elastic modulus of the theoretical calculations and experimental measurements are 1.05–1.24 TPa and 1 TPa, respectively [7,8,9]. The thermal conductivity (TC) of graphene is up to 5300 Wm^−1^ K^−1^ [10]. At room temperature, the carrier mobility of graphene is as high as 15,000 cm^2^ V^−1^ s^−1^ [2], and its carrier mobility is not affected by temperatures in the range of 10–100 K, which proves that defect scattering is the main scattering mechanism of the electrons in graphene [11]. The light absorption of single-layer graphene is only 2.3% [12,13,14], and it has excellent nonlinear optical properties [15]. Graphene also has exceptional magnetic and spintronic properties [16].

## 2. The Crystal Structure and Electronic Structure of Graphene

The ideal graphene is the thinnest two-dimensional crystal (0.35 nm). The bond length between C and C is 0.142 nm ($a_{c-c}$). In the crystal structure of graphene, each cell contains two C atoms, A and B. Carbon atom A and atom B are not equivalent; see Figure 1b. The lattice constant is 2.460 Å [17]. Each C atom has four valence electrons, three of which form three σ bonds in *sp^2^* orbital hybridization with neighboring C atoms, respectively, in the graphene plane. This regular hexagonal crystal structure makes graphene’s planar structure extremely stable. The tensile elastic modulus of graphene is as high as 1 TPa and the tensile strength is as high as 130 GPa [7,8,9]. The structure of graphene remains stable when external forces are applied to it; the other electron in the *P* orbital contributes to the nonlocalized π and π^*^ bonds, which form the highest occupied molecular orbital (HOMO) and lowest unoccupied molecular orbital (LUMO). The π and π^*^ bonds degenerate at point K in the Brillouin zone (BZ) of graphene; see Figure 1c. The Fermi surface shrinks to a point, forming a bandwidth-free metal-like band structure [18,19,20,21], which gives graphene extremely high carrier mobility.

Researchers have described the interactions between graphene’s π electrons using a tightly bound model [3,18,20]:

(1) $$\varepsilon^{\pm }(k_{x},k_{y})=\pm \gamma_{0}\sqrt{1+4\cos\frac{\sqrt{3}k_{x}a}{2}\cos\frac{k_{y}a}{2}+4\cos^{2}\frac{k_{y}a}{2}}$$

where the value of a is $\sqrt{3}a_{c-c}$ and $\gamma_{0}$ is the transfer integral that corresponds to the matrix element between π orbitals of adjacent carbon atoms, with the magnitude ranging from 2.8 eV to 3.1 eV. The plus energy of Equation (1) corresponds to the π^*^ band, and the minus energy corresponds to the π bond, respectively. Figure 1d illustrates the electronic dispersion in the honeycomb lattice, which is the electronic structure of monolayer graphene. Since the electronic states near the Dirac points (DPs) are composed of different sublattices, the dual component wave function corresponds to their relative contributions. The Schrödinger equation corresponding to these electrons is as follows [2]:

(2) $$\hat{H}=\hbar v_{F}\left(\begin{array}{cc} 0 & k_{x}-ik_{y}\\ k_{x}+ik_{y} & 0 \end{array}\right)=\hbar v_{F}\vec{\sigma }•\vec{k}$$

The Fermi group velocity corresponds to $v_{F}\approx 1\times 10^{6}ms^{-1}$, $\vec{\sigma }$ corresponds to the Pauli matrix, and $\vec{k}$ corresponds to the momentum of graphene’s quasiparticles. Figure 1e–g shows the electronic structures of single-layer graphene and the electronic structure of double-layer graphene in the AA and AB stacking modes.

Graphene’s unique physical and chemical properties are closely related to its electronic energy band structure. The energy level distribution of graphene can be calculated based on independent C atoms and the potential generated by the surrounding C atoms as perturbation. Spread out near the DPs, the energy is linearly related to the wave vector (similar to the dispersion of photons), and a singularity occurs at DPs. This means that the effective mass of the electrons in graphene is zero near the Fermi energy level, which also explains the material’s exceptional electrical properties.

## 3. Graphene’s Novel Electronic Properties

Graphene’s exclusive crystal and electronic structures give it many novel and inimitable physical properties, including anomalous quantum hall effects (QHEs) [20,22,23], ambipolar electric field effects (AEFEs) [24], Klein tunneling (KT) [25,26], and ballistic transport (BT) [27,28]. With further research on graphene nanomaterials, more electrical characteristics have been discovered and recognized.

### 3.1. Current Vortices, Electron Viscosity, and Negative Nonlocal Resistance

Electron transport properties are similar to those of viscous fluid in strongly related electronics systems, known as the quantum critical effect, which is a typical collision-controlled mass transfer characteristic. However, the study of this phenomenon has been hindered by the lack of macroscopic features of electron viscosity. Levitov et al. determined the vortex characteristics associated with the easy verification of the macroscopic mass transfer performance [29,30,31,32]. The eddy current generated by viscous flow can be applied to the field of the opposite driving current, which results in a negative nonlocal voltage. This suggests that the latter plays an important role in the superconducting zero resistance viscosity system. In addition to providing a diagnosis of viscous mass transfer from the ohmic flow, the electrical response of the signal changes, providing a powerful way to directly measure the viscosity and resistivity ratios. The intense electron hole plasma interaction in high-mobility graphene provides an exclusive connection between the critical quantum electron transport and many hydrodynamic phenomena.

**Figure 2:**
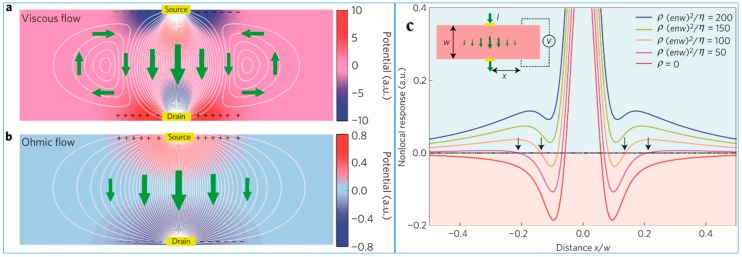
Current streamlines and potential map for viscous and ohmic flows. White lines show current streamlines, colors show electrical potential, and arrows show the direction of current. (**a**) Mechanism of a negative electrical response: viscous shear flow generates vorticity and a backflow on the side of the main current path, which leads to charge buildup of the sign opposing the flow and results in a negative nonlocal voltage. (**b**) In contrast, ohmic current flows down the potential gradient, producing a nonlocal voltage in the flow direction. (**c**) Nonlocal response for different resistivity-to-viscosity ratios,ρ/η [33]. Copyright 2016, Springer Nature.

It has been a prominent problem that the zero resistance of the viscous flow is related to superconductivity. The viscous flow caused by the vortex leads to the unique macroscopic transport behavior. Levitov et al. predicted that the vorticity of the shear flow with viscosity may cause the return current [33], which is opposite to the applied field. Figure 2a,b shows a negative signature of the nonlocal voltage, which provides a clear signature of collective viscosity. The characteristic symbols associated with this behavior change the spatial pattern of the potential, as shown in Figure 2, which can be observed directly using modern scanning microscopy techniques [34].

Figure 2a,b shows the basic properties of the negative response from the shear flow. The collective behavior of the viscous system results from the momentum exchange of the fast exchange carrier while preserving the conservation of net momentum. The momentum is still referred to as a conserved quantity as it will produce a hydrodynamic momentum transfer mode; that is, the momentum flows in space, in a transverse diffusion to the source drain current and away from the nominal current path. As a result of this process, the shear flow is established to produce vorticity and the application field of the (noncompressible fluid) opposite reflux direction. The direct effect of such a complex and apparently noncurrent mode on the electrical response produces a reverse electric field acting on the opposite field driving the source drain current; see Figure 2c. This results in the presence of a negative nonlocal resistance, even in the presence of quite significant ohmic flow; see Figure 2c.

### 3.2. Transition Between Electrons and Photos

Graphene’s inimitable electronic properties make it a new infrared frequency domain plasmon waveguide and terahertz metamaterial [35,36,37,38,39,40,41,42,43,44,45,46,47,48,49]. By excitation of photons or electrons, the collective oscillations of electrons on the surface of a conductor is the surface plasmon. When photons and graphene’s surface plasmon are coupled, they form surface plasmon polaritons (SPPs). As an alternative to conventional metal plasmon, graphene surface plasmons (GSPs) can be used for optical metamaterials [40], optical absorption, and optical conversion [41,42].

**Figure 3:**
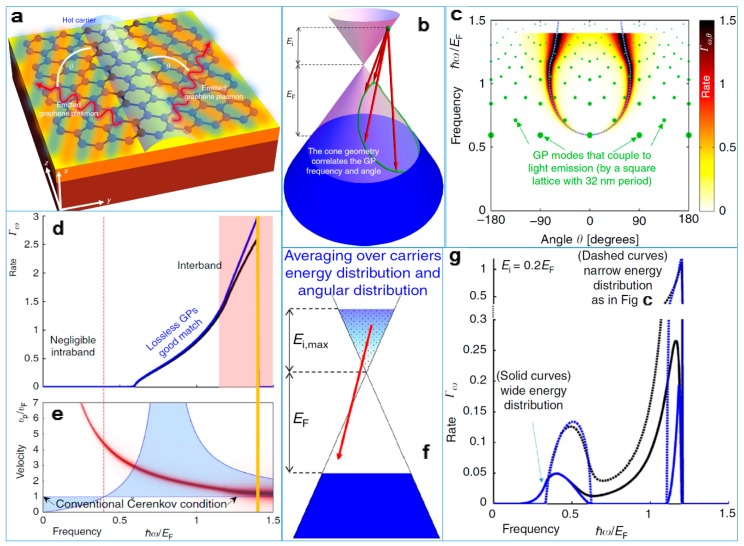
(**a**) Illustration of the plasmon emission from charge carriers in graphene via a 2D Čerenkov process. (**b**) Illustration of the possible transitions. The hot carrier (green dot) has a range of potential transitions (red arrows) with distinct final states (green curves and circles), emitting plasmons that satisfy conservation of momentum and energy (corresponding to the height and angle of the red arrows). In this way, the cone geometry correlates the graphene surface plasmon (GSP) frequency and angle. The projection of these arrows to a 2D plane predicts the in-plane angle y of the plasmonic emission, matching the (**c**) map of GSP emission rate as a function of frequency and angle. (**d**) Spectrum of the ČE (Čerenkov effects) GSP emission process of radiation rate. (**e**) Explaining the GSP emission with the quantum ČE. The GSP phase velocity is plotted as a red curve, with its thickness presenting the GSP loss. (**f**) Illustration of the distribution of hot carriers, which is taken to be an exponential multiplied by the linear electron density of states in graphene. The exponential decay is (**g**) $e^{E_{1}/0.01eV}$ with maximum hot carrier energy of $E_{i,\max}=0.2eV$ corresponding to (d) [48]. Copyright 2016, Springer Nature.

Researchers have found that when light hits a single layer of graphene, it can slow down by hundreds of times [48,49]. Researchers have studied the hot carriers located inside graphene, which form GSPs on graphene’s surface; see Figure 3a. Figure 3b–g shows the test results and analysis of the experimental results (The GP in the figure stands for graphene plasmon.). Slowing photons (particles of light) travel through a single layer of graphene much as electrons do through the same material. Electrons travel very fast in a single layer of graphene, at a million meters per second, about one-third the speed of light in a vacuum. The speed of the two particles is close enough to strongly interact. When the material is adjusted to match the speed of the two particles, graphene slows the speed of the photons and the rapid movement of the electrons across a graphene surface. This suggests that graphene can be used to create a possible intrinsic effect, producing light instead of capturing it. The theoretical research suggests this could generate light by a new way. This conversion is possible because electrons’ drift velocity in graphene is close to light, breaking the “light barrier” just as shock waves produce sound when breaking the “sound barrier”. In graphene, this will result in a shock wave of light on a two-dimensional level.

This new approach may eventually become more efficient and adjustable. Highly efficient and controllable plasmon generation properties that are compatible with current microchip technology are a new method of creating core chips for optical circuits, which is a new research direction in the evolution of computer technology toward smaller and more efficient devices.

### 3.3. Electron Transport Properties in Nitrogen-Doped Graphene

The presence of impurities, especially charged impurities in a lattice, can affect graphene’s electrical properties [50,51,52,53,54]. When approaching the DPs, due to the loss of state density, graphene’s transport properties are highly sensitive to the scattering of charged impurities. Therefore, physical and chemical doping of graphene can be used to improve its electrical properties. Among many dopants, both theories and experiments have shown that an n-doped graphene lattice can realize n-doping and the carrier can maintain high mobility [55].

**Figure 4:**
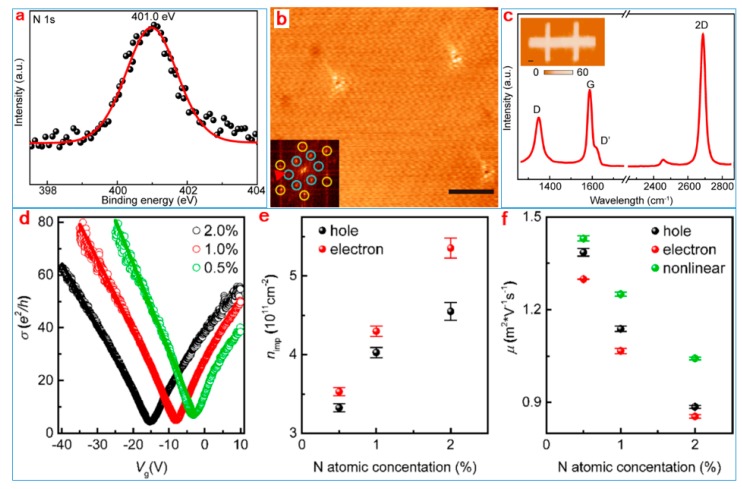
(**a**) N 1s core-level XPS (X-ray photoelectron spectroscopy) spectra of an as-formed graphitic N-doped (2% N atomic concentration) graphene film. (**b**) Scanning tunneling microscope (STM) image of an as-formed graphitic N-doped graphene sample ($V_{bias}=-10mV$ and $I_{set}=100pA$). (**c**) Raman spectra of a graphitic N-doped (2% N atomic concentration) graphene film (The letters on each Raman peak in the figure indicate the vibrational symmetry of the Raman peak.). Transport properties (conductivity (**d**), carrier concentration (**e**) and mobility (**f**)) of as-formed N-doped graphene films [55]. Copyright 2017, American Chemical Society.

High-quality nitrogen-doped graphene is prepared using chemical vapor deposition (CVD) methods. The spectral line in Figure 4a indicates that there is a characteristic peak at 0 eV, and it was confirmed that the doped nitrogen was graphitized nitrogen, in which the doping concentration was 2.0%. The doping of nitrogen atoms between graphene lattices was demonstrated using a scanning tunneling microscope (STM) graph. The white region in Figure 4b represents the location of nitrogen doping. In the figure, the color was caused by the intervalley scattering in the graphene lattice caused by single doping. The illustration is the fast Fourier transform (FTF) corresponding to the STM. In Figure 4c, the peak height of the D peak in the Raman spectrum increased with the increase in nitrogen doping. The 2D peak indicates that the nitrogen-doped graphene prepared via the experiment had good crystallization. The diagram is a test sample image. Figure 4d shows the relationship between the conductivity σ of nitrogen-doped graphene with different doping concentrations and the grid voltage ***V***_g_ at temperature (*T*) = 9 K. Solid lines of different colors represent different doping concentrations. As shown in the figure, the negative location of the DPs is due to the sample’s n-type doping characteristics, and as the nitrogen doping concentration increases, the DPs moves toward negative values. Figure 4e shows the evolution of the charge impurity density with the nitrogen doping concentration. Figure 4f shows that after the introduction of more scattering centers, carrier mobility in nitrogen-doped graphene decreases as the nitrogen doping concentration increases.

The test results confirmed the electron hole valley scattering asymmetry of the charge transport property of the nitrogen-doped graphene. Valley electron scattering is much stronger than hole scattering, and the scattering rate increases with the increase in the nitrogen doping concentration. This is because the nitrogen-doped graphene forms a positive center, causing large-angle scattering for electron transport. Therefore, graphene carrier scattering can be effectively regulated by adding different amounts of graphene nitrogen to control graphene’s electrical characteristics.

### 3.4. Strong Current Tolerance

Graphene can also withstand very high currents and quickly balance out missing charges. Researchers conducted an experiment to prove that the electrons in graphene are extremely mobile and react very quickly. Collisions of Xe^+^ with the supercharged charges on graphene films can detach many electrons from a very precise point [56]. Within a few femtoseconds, graphene can quickly resupply the electrons. This will lead to the appearance of ultrastrong currents that cannot be sustained under normal circumstances. These unusual electrical properties make graphene a potential candidate for future electronic applications.

**Figure 5:**
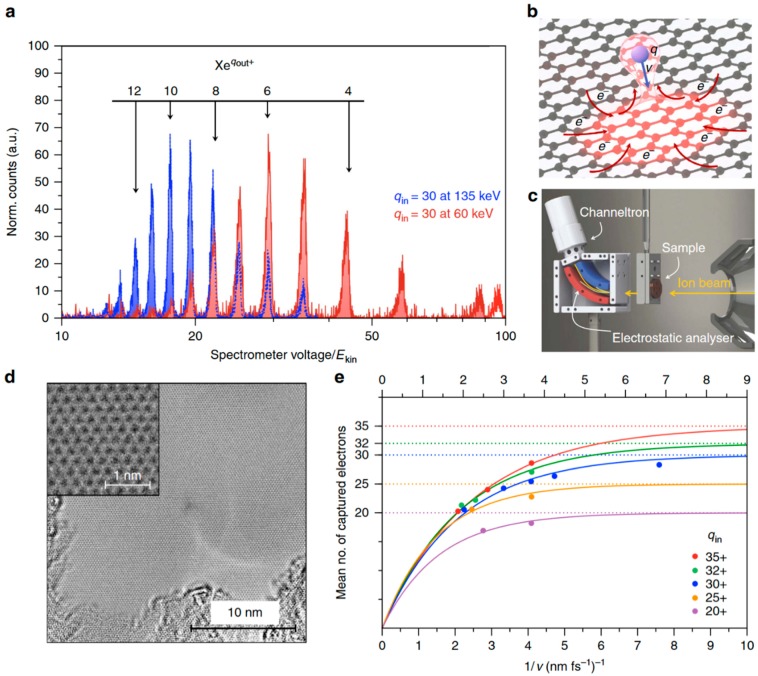
Experimental scheme and results. (**a**) Measured spectra of a Xe^30+^ beam at kinetic energies of 135 and 60 keV (blue and red, respectively) transmitted through a freestanding single-layer graphene (SLG) sheet with different amount of charge injected (*q_in_*). Exit charge states $q_{out}$ are calculated from the spectrometer voltage of the electrostatic analyzer. The exit charge state distribution shifts towards smaller average exit charge ${\bar{q}}_{out}$ for slower ions. (**b**) Schematic of the interaction process between freestanding SLG and an approaching highly charged ion (HCI). The HCI extracts a lot of charge from a very limited area on the femtosecond time scale, leading to a temporary charge-up of the impact region (*q* is the amount of electricity, and v is the speed.). (**c**) Sketch of the experimental setup with the target holder and electrostatic analyzer. (**d**) TEM image of a freestanding monolayer of graphene after irradiation with Xe^40+^ ions at 180 keV with an applied fluence of 10^12^ ions per cm^2^ (about six impacts on the shown scale). No holes or nanosized topographic defects could be observed. (**e**) Average number of captured and stabilized electrons ($q_{in}-{\bar{q}}_{out}$) after transmission of $Xe^{q_{in}+}$ ions through a single layer of graphene as a function of the inverse projectile velocity for different incident charge states [56]. Copyright 2016, Springer Nature.

Researchers used a single xenon ion to travel through the graphene layer, so that each xenon ion carried approximately 20 electrons through the graphene region. At this point, the C atom with a missing electron is squeezed out of the graphene, and the location of the missing electron is immediately replenished by other electrons, which lasts only a matter of femtoseconds. This means there is a large amount of electron flow in a short period of time; that is, a high density of current is transmitted on graphene. Figure 5 shows a schematic diagram of the experimental equipment and test results. Strong currents from areas adjacent to the graphene membrane quickly replenish the electrons before the positive charges repel each other and cause an explosion. To accomplish this, graphene must carry a current density approximately 1000 times higher than any material would normally tolerate. The surrounding current density is 1000 times that of a normally destructible material, but graphene can withstand these extreme currents with no damage. The movement of extremely high charges on graphene is of great significance for a range of potential applications, and graphene may be used in superfast electronics in the future.

### 3.5. Novel Electrical Properties of Graphene/Graphene van der Waals Heterostructure

A van der Waals (VdW) heterostructure is a vertical stack of two-dimensional materials. Based on the rich functionality of two-dimensional materials, more engineering manipulation can be realized to obtain unexpected new characteristics. The graphene-based van der Waals heterostructure is introduced as a substrate from h-BN (hexagonal Boron Nitride). The peculiar physical and chemical characteristics of the heterogeneous structure have provoked much interest. Of the many VdW heterostructures based on graphene (graphene/graphene, graphene/h-BN, graphene/MoS_2_, and graphene/Si, among others), VdW heterostructures produced by double layers of graphene are particularly interesting. Because the lattices of the two layers correspond precisely, the physical and chemical properties are equivalent [57,58,59,60,61]. Novel electrical properties are produced in the superlattices of graphene/graphene heterostructures [62,63,64].

**Figure 6:**
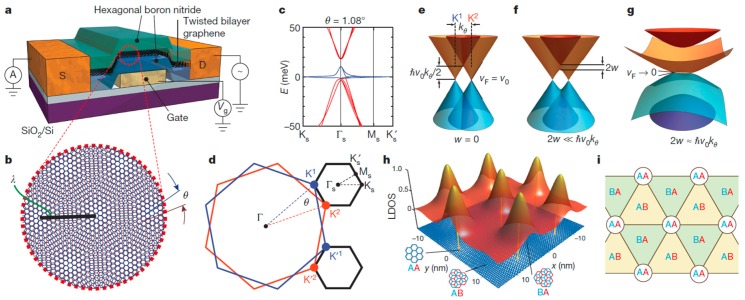
(**a**) Schematic diagram of twisted double graphene devices. (**b**) Moiré superlattice in graphene/graphene heterostructure. (**c**) The band structure of the double graphene at an angle of *θ* = 1.08°. (d) Mini-BZ in graphene/graphene heterostructure. (**e**–**g**) A schematic diagram of overlapping bands in different energy regions. Localized density of states (LDOS) (**h**) of heterogeneous structural regions in different stacking modes(**i**). [62]. Copyright 2018, Springer Nature.

Researchers from the Massachusetts Institute of Technology (MIT) have found that the electron properties of vertically stacked graphene heterostructures are closely related to the arrangement of C atoms in graphene [62]. The different arrangement of atoms also affects the movement of electrons between the layers. In general, electrical behavior is dominated by energy, and the energy of electrons involved in the movement of electrons between atoms within a single layer of graphene is on an order of magnitude of eV, while the energy involved in the movement of electrons within a double layer of graphene is on an order of magnitude of several hundred meV. Figure 6 shows a diagram of experimental equipment and calculation results [62].

**Figure 7:**
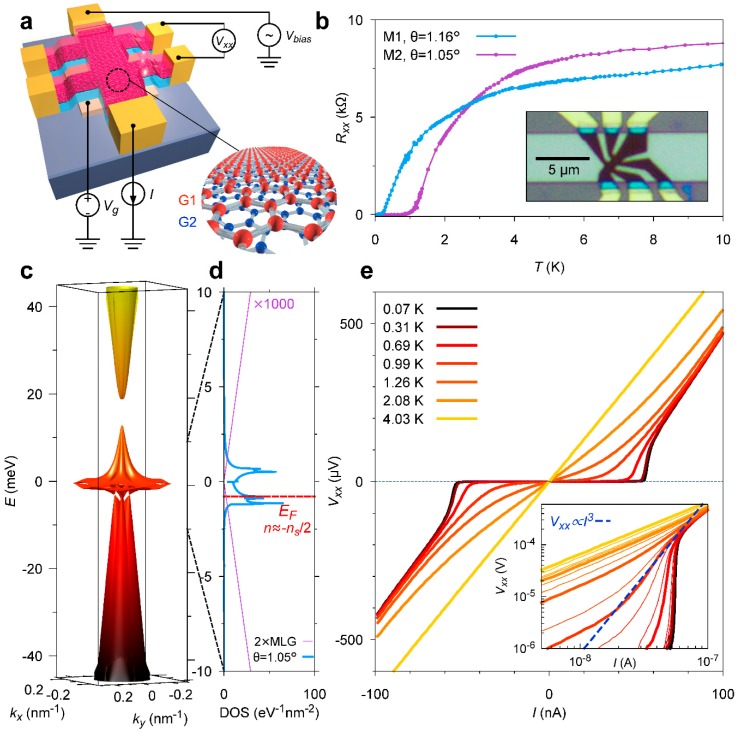
2D superconductivity in a graphene superlattice. (**a**) Schematic of a typical twisted bilayer graphene (TwBLG) device and four-probe measurement scheme. The stack consists of top h-BN, rotated graphene bilayers (G1, G2), and bottom h-BN. (**b**) Measured four-probe resistance ($R_{xx}=V_{xx}/I$) in two devices M1 and M2, with twist angles *θ* = 1.16° and *θ* = 1.05°, respectively. (**c**) The band structure of TBG at *θ* = 1.05° in the first mini-Brillouin zone (MBZ) of the superlattices. (**d**) The density of states (DOS) corresponding to the bands shown in (**c**), zoomed in to −10–10 meV. (**e**) *I–V* curves for device M2 measured at *n* = −1.44 × 10^12^ cm^−2^ and various temperatures. At the lowest temperature of 70 mK, the *I–V* curve shows a critical current of approximately 50 nA [63]. Copyright 2018, Springer Nature.

For graphene with a highly ordered structure, the electrical properties depend on symmetry. The MIT researchers created a rotating, twisted, two-layer graphene heterostructure that controls the electron state of the entire system through interactions between the electrons. The dislocation caused by rotation misaligns the electron band structure in the graphene layer and increases the single cell size. Twisted bilayer graphene (TwBLG) produces two new electron states [63,64]. One electronic state is the Mott insulated body that results from the strong repulsion between the electrons. The other is the superconducting state that results from the strong attraction between electrons to produce zero resistance. When the small rotation angle approaches the magic angle (<1.05°), distortion of the vertical stack of atoms in the double-layer graphene area will form a narrow electronic band, enhancing the electronic interaction effect and resulting in a nonconductive Mott insulation state. In a Mott insulation state, a small charge carrier can be successfully converted into a superconducting state. Figure 7 shows the test results of superconductivity in a graphene/graphene heterostructure [63].

The MIT researchers created TwBLG that controls the electron states of the entire system through interactions between electrons. The dislocation caused by rotation misaligns the electron band structure in the graphene layer and increases the single cell size. The study found that in stacked graphene layers, electrical behavior is very sensitive to the arrangement of atoms, affecting the movement of electrons between layers.

### 3.6. The Interaction between Plasmons and Electrons in Graphene

The surface plasmons of 2D nanomaterials have distinctive electrical properties, and applications such as the interaction between plasmons and electrons and catalytic reactions are also well known, especially in surface plasmons based on grapheme [65,66,67,68,69,70].

**Figure 8:**
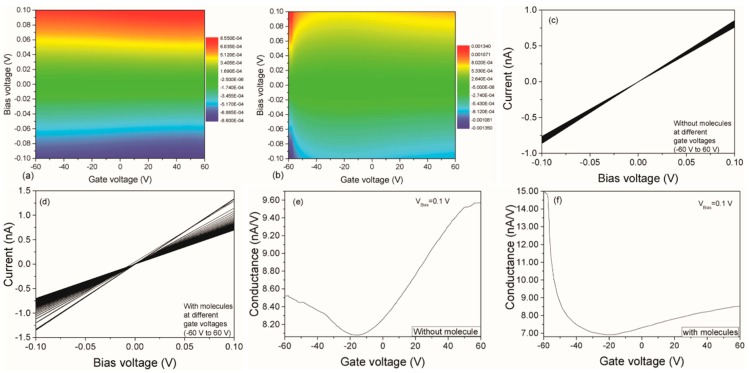
Gate- and bias-voltage-dependent electrical current for a device without (**a**) and with (**b**) addition of molecules; (**c**,**d**) the current varies with bias voltage at different gate voltages; (**e**,**f**) gate-voltage-dependent conductance for a device without and with molecules, where $V_{Bias}=0.1\;V$ [69]. Copyright 2017, Wiley.

Cao et al. reported on a plasmon–exciton coupling device [69]. They first prepared a complementary metal-oxide semiconductor (CMOS)-like device composed of gold dots and graphene. The device can easily be added to the gate voltage or can inject current into the device through the source and drain electrodes. They included a series of studies on the electrical properties of plasma–exciton-coupled devices under different gate voltages and source–drain voltages. The source–drain bias voltages at different gate voltages were measured before and after the catalyzed molecules were placed in the center of the device. The source–drain bias increased at the same gate voltage after the molecules were placed and showed varying bias change behaviors at different gate voltages; see Figure 8a,b. This occurred because the chemical potential of graphene is regulated by the gate voltage. Figure 8c,d shows the operation of the device current as the bias voltage changed. First, the volt–ampere characteristics of the bias voltage and current showed a perfect linear relationship. Second, the volt–ampere characteristic curve (slope) changed little at different gate voltages when no molecules were added to the device. When there was no molecule, the gate voltage had little ability to regulate the chemical potential of graphene. Third, after the device was added to the molecule, the slope of the volt–ampere characteristic curve was very different, indicating that the gate voltage had an increased regulation range for the device’s chemical potential. These phenomena indicate that charge transfer occurs between the molecule and graphene and that the gate voltage also regulates the charge transfer. This can also be concluded by the relationship between the gate voltage and conductivity, as shown in Figure 8e,f. Focusing on the relationship between the volt–ampere characteristic curve and the gate voltage, the gate voltage can be regulated in both positive and negative directions for charge transfer. This is a very good property for photooxidative reduction catalysis. Since the oxidation reaction requires holes, the reduction reaction requires electrons. Such devices provide free holes or electrons for the reaction through different gate voltage controls.

## 4. The Thermal and Thermoelectric Properties of Graphene

### 4.1. The Thermal Conductivity (TC) Measurement of Graphene

**Figure 9:**
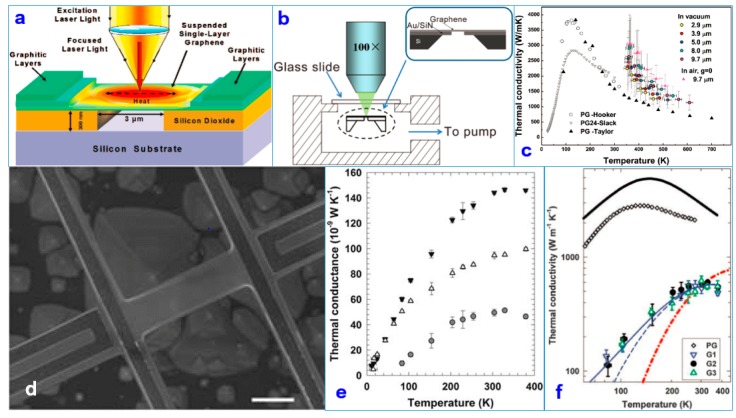
The thermal conductivity (TC) of graphene. (**a**) Schematic of the experiment showing the excitation laser light focused on a graphene layer suspended across a trench [71]. Copyright 2008, American Chemical Society. (**b**) Schematic of the experimental setup for thermal transport measurement of suspended graphene [72]. (**c**) Thermal conductivity of the suspended chemical vapor-deposited (CVD) graphene as a function of the measured temperature of the graphene monolayer suspended in a vacuum over holes of various diameters [72]. Copyright 2011, American Chemical Society. (**d**) SEM image of the suspended device, the central beam, and the folded edge of the SLG ribbon near the right electrode. (**e**) Measured thermal conductance of G2 before (solid downward triangles) and after (unfilled upward triangles) the SLG was etched, with the difference being the contribution from the SLG (circles). (**f**) The relation diagram of thermal conductivity of different samples with temperature change [73]. Copyright 2010, Science.

In 2008, researchers used confocal micro-Raman spectroscopy to measure the heat transport of suspended graphene nanoribbons [71,72]. They found that the thermal conductivity (κ, TC) of SLG reached 3500–5300 W/mK, and the mean free path of the phonons was 775 nm at room temperature [71]. The device is shown in Figure 9a. The experimental results confirmed that the heat transfer of graphene mainly comes from the phonon contribution. The same study was conducted again in 2011. To be immune to air heat transfer, researchers measured suspended graphene at the pore size of 2.9–9.7 μm in a vacuum environment to eliminate the loss of ambient air heat. Figure 9b shows the device. The TC of graphene was measured up to (2.6 ± 0.9)–(3.1 ± 1.0) × 103 Wm^−1^ K^−1^ at a temperature of 350 K. The experimental device configuration is shown in Figure 9b near the TC of graphene up to (2.6 ± 0.9)–(3.1 ± 1.0) × 10^3^ Wm^−1^ K^−1^ [72].

The phonon transmission mode and scattering mechanism of graphene’s thermal conductivity have a significant impact. Numerous studies have shown that the substrate of graphene has an unavoidable effect on its thermal conductivity. After the two-dimensional material contacts the substrate, the thermal conductivity normally decreases significantly after the two-dimensional material contacts the substrate because the thermal conduction is mainly determined by the phonon conduction. When the graphene comes into contact with the substrate [73,74,75], the surface or edge of the graphene is very sensitive. In 2010, researchers measured the TC of graphene deposited on silica substrates and found that it had been reduced due to scattering at the substrate interface, but it still reached 1000 Wm^−1^ K^−1^ [73]. Figure 9c shows the thermal conductivity of the suspended CVD graphene as a function of the measured temperature of the graphene monolayer suspended in a vacuum over holes of various diameters. Figure 9d shows a STM representation of the graphene sample. The thermal properties of graphene nanostructures may play a very important role in future nanodevices. Figure 9e shows a comparison between the thermal conductivity of graphene before and after etching and that of single-layer graphene. Figure 9f shows the relation diagram of thermal conductivity of different samples with temperature changes.

It is apparent from the measurement of the thermal conductivity of graphene that all factors, such as the shape and thickness, affect graphene’s thermal conductivity. In general, the size of the graphene in the heat transfer direction is positively correlated with the thermal conductivity. In general, the larger the linear size of graphene, the higher the thermal conductivity; when graphene is on the substrate, the substrate has a great influence on the thermal conductivity. This is because the interaction between the graphene and the substrate leads to changes in the graphene’s lattice constant. The temperature also affects the magnitude of the TC.

### 4.2. Length-Dependent and Temperature-Dependent TC of Graphene

Based on the relationship between the TC and length, researchers also specifically analyzed the relationship between the TC and the temperature [75]. The results indicate that all of the graphene nanoribbons exhibit the same properties as the temperature. Thermal conductance (σ) adds to the platform as the temperature increases. The TC (κ) increases with the length of the graphene nanoribbons over the entire temperature range.

**Figure 10:**
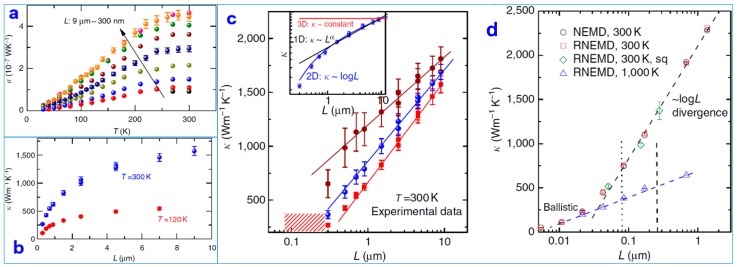
(**a**) The variation in the thermal conductance (σ) of a graphene nanoribbon with temperature (T) over a length of 9 μm to 300 nm. (**b**) Image of the TC (κ) with length (L) at temperatures of 120 K and 300 K. ***R***_total_ is the thermal contact thermal resistance. Experimental (**c**) and various simulation methods (**d**) results on length-dependent thermal conductivity [76]. Copyright 2014, Springer Nature.

Earlier sections of this article cited examples of theoretical calculations for the TC of graphene [76]. However, the role of graphene in the ZA mode during heat transfer remains controversial. In particular, the solution to the Peierls–Boltzmann equation shows that the ZA mode is the main heat carrier for graphene transport and has a strong dimensional dependence [77,78,79]. Research conducted through experimental data and nonequilibrium molecular dynamics (NEMD) simulation resulted in the perfect combination [80,81]. Figure 10c shows the relationship between the TC (κ) and the length (L) [75]. The value of κ increased with the increase in L at a temperature of 300 K. The temperature did not saturate in the longest *L* = 9 μm of graphene. *k* was proportional to logL at L > 700 nm. This is because the length of graphene is comparable to the phonon mean free path. The researchers also observed that the size far exceeded the ballistic length at 1000 K and at 300 K; see Figure 10d. After comparing the different aspect ratios of the graphene nanoribbons, their simulated results agree with Figure 10d. The relationship between the TC and the length of the single-layer graphene nanoribbons has been revealed experimentally.

The researchers used experimental measurements and nonequilibrium molecular dynamics to simulate the thermal conduction of a single layer of suspended graphene as a function of the temperature and sample length. Different from bulk materials, the thermal conductivity continues to increase at 300 K. Although the length of the sample is much larger than the mean free path of the phonons, the thermal conductivity remains logarithmically discrete with the length of the sample. This is the result of the two-dimensional properties of phonons in graphene, which provides a basis for understanding heat transfer in two-dimensional materials.

### 4.3. Influence of Boundary or Configuration on Thermal Property and Thermal Rectification Effect

Many theoretical studies and experiments have shown that the different boundaries and configurations of graphene nanoribbons have a certain impact on the TC [82,83,84,85,86,87,88,89,90,91,92,93].

**Figure 11:**
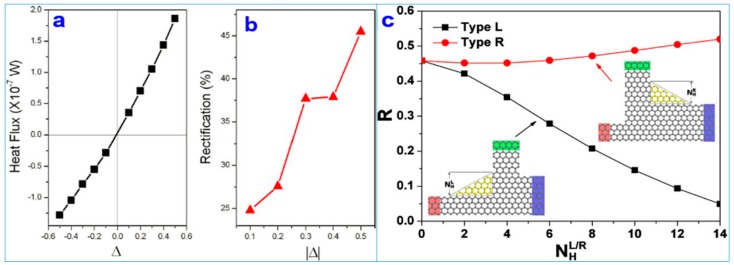
(**a**) Heat flux (*J*) versus ∆; (**b**) Rectifications versus ∆ for the single-layer graphene Y junctions [84]. Copyright 2011, Royal Society of Chemistry. (**c**) The rectification ratio R versus the side height of corner $N_{H}^{L/R}$ for the two types of asymmetric three-terminal graphene nanojunctions (TGNJs) with armchair-edged corners [85]. Copyright 2011, American Physical Society.

The TC of zigzag-type graphene nanoribbons is 30% higher than that of armchair-type graphene nanoribbons at room temperature, indicating obvious heat transport anisotropy [82]. Figure 11a,b demonstrates that the heat flux of Y-type graphene nanoribbons studied via molecular dynamic (MD) simulation shows a significant thermal rectification effect. Moreover, the bilayer Y-type nanoribbons can achieve a larger rectification ratio due to the interaction between the layers than the single-layered Y-type nanoribbons, thus providing a theoretical basis for the thermal management of nanoelectronics [84]. Figure 11c shows the phonon properties of three-terminal graphene nanojunctions (GNJs) [85]. This study demonstrates that the transport direction of the heat flux moves along the narrow end to the wide end and produces an obvious ballistic thermal rectification effect. The thermal rectification ratio of 200% is dependent on the asymmetry of the nanojunction, indicating excellent ballistic heat transport properties, making the preparation of nanodevices possible.

### 4.4. The Effect of Atomic Edge Variation and Size Change on TC

**Figure 12:**
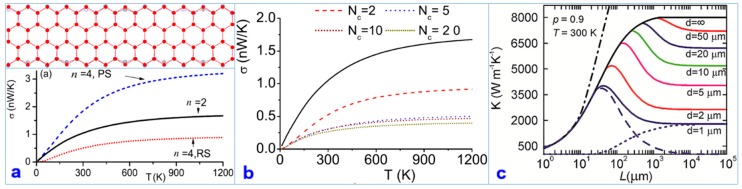
(**a**) Transport properties of graphene nanoribbons (GNRs) with width *n* = 2 and *n* = 4. Dotted curve corresponds to the case for the relaxed structure (RS) of GNR-4 and the dashed curve corresponds to the case for the perfect structure (PS) of GNR-4. Full circles represent the configuration of the perfect GNR-4. Circles with and without “+” represent two configurations after relaxation. (**b**) Thermal conductance of the sandwiched device versus temperature for different numbers of cells *N*_c_ in the central region. For comparison, the solid line corresponds to the conductance of a GNR-2 of infinite length [94]. Copyright 2009, American Physical Society. (**c**) Dependence of the thermal conductivity of the rectangular graphene ribbon on the ribbon length L shown for different ribbon width ***d***. The specular parameter is fixed at *p* = 0.9 [95]. Copyright 2012, American Chemical Society.

Using the nonequilibrium green function (NEGF) method combined with first-principles calculations, researchers revealed the influence of the edge atom position on the TC of graphene nanoribbons [94]. Figure 12a,b presents the results of this study. When the width of the nanoribbons is *n* > 12, the TC possesses obvious quantization characteristics and is independent of the nanoribbon width. When the nanoribbon width is 2 < *n* < 12, due to the obvious boundary effect of the nanoribbons, the quantum heat transport is destroyed.

Another theoretical simulation revealed the dependence of the TC on the length of graphene [95]. Researchers simulated the relationship between the TC and the lateral dimension of graphene nanoribbons and again revealed the mean free path of acoustic phonons and the nonmonotonic dependence of the nanoribbon length *L*. Moreover, past studies reported that the bulk three-dimensional phonons in graphene greatly contribute to the TC in the graphene plane. Figure 12c shows the results.

Many theoretical calculations and experiments have revealed that graphene’s TC can be changed through the hybridization of grapheme [96], adsorption of metal atoms [96,97], gradient surface hydrogenation [76,98], grain size engineering [99], fluorination [100], carbon isotope doping [101], vacancies and defects engineering [102,103], and so on.

### 4.5. The Thermoelectric Properties of Graphene

The performance of thermoelectric materials is generally measured by the thermoelectric optimal value *ZT* (the thermoelectric figure of merit) [58,104]:

(3) $$ZT=\sigma S^{2}T/\left(\kappa_{e},\kappa_{ph}\right)$$

where σ is the electronic conductivity, *S* is the Seebeck coefficient, $\kappa_{e}$ and $\kappa_{ph}$ are the electronic thermal conductance and phonon thermal conductance, respectively, and *T* is the average temperature of the device. The formula indicates that the higher the *ZT*, the higher the conversion efficiency between the device’s thermal energy and electric energy. Outstanding thermoelectric materials must have higher Seebeck coefficients, good electrical conductivity, and very low thermal conductivity. Therefore, it has become a focus of research to identify thermoelectric materials with higher Seebeck coefficient, improve their electronic conductivity, and reduce their thermal conductivity [105,106,107].

#### 4.5.1. Thermoelectric Properties in Graphene Nanoribbons (GNRs)

**Figure 13:**
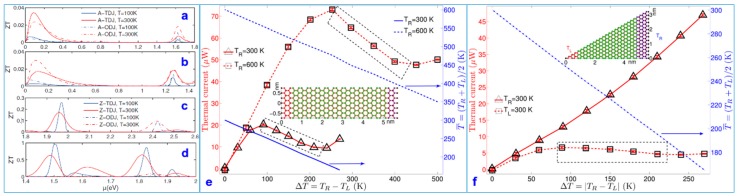
The figure of merit *ZT* at the two different temperatures of 100 K and 300 K in (**a**,**b**) for armchair edge junctions and (**c**,**d**) for zigzag edge junctions. Thermal current (left vertical axis) and average temperature (right vertical axis) vs. temperature difference ∆*T* [106]. Copyright 2012, American Physical Society. The dashed boxes highlight negative differential thermal conductance (NDTC) in (**e**). The inset shows the structure of the GNR (1.5 nm × 6 nm). (**f**) Thermal current (left vertical axis) and average temperature (right vertical axis) vs. temperature difference ∆*T* in triangular GNRs is shown in the inset. The dashed box highlights NDTC [107]. Copyright 2011, American Physical Society.

Thermoelectric conversion is currently a popular field of research. Researchers studied the ballistic thermoelectric properties of graphene nanoribbon-based heterojunctions [106]. The results reveal that the binding structure affects the transport of electrons, whereas the fluctuations in electrons are strongly enhanced by the thermoelectric power. The first four panels in Figure 13 show the *ZT* at *T* = 300 K and the *ZT* at *T* = 100 K for two different graphene edge heterojunctions. The calculation of the ballistic thermoelectric properties based on graphene nanoribbons heterostructures using the nonequilibrium green function and the Landau transport theory provides a method for understanding the thermoelectric properties of graphene. The phonon heat conduction under the influence of different heterostructures was basically similar, but the influence of the heterostructure geometry and geometric details on electron transport was substantial, and the change in the electronic transport greatly enhanced the thermoelectric properties. These parameters further improve the thermoelectric properties of nanomaterials.

The nonlinear thermoelectric properties of triangular graphene nanoribbons at the armchair boundaries of armchair-GNR (AGNR) and zigzag-GNR (ZGNR) were also modeled by MD [107]. Researchers observed that negative differential thermal conductance (NDTC) appeared in the system when GNRs had large temperature deviations and beyond the range of the linear influence, and the NDTC could be controlled by the temperature. For rectangular GNRs, as the GNR length increases, the NDTC gradually decreases and eventually disappears; see Figure 13e. For triangular GNRs, the NDTC only exists when heat flows from the narrow end to the wide end; see Figure 13f. These results provide theoretical basis for the thermal management and thermal signal processing of nanomaterials.

#### 4.5.2. Thermoelectric Spin Voltage (TSV) in Graphene

Spin-dependent thermal effects, or the interaction between spin and thermal current, have been demonstrated in ferromagnetic materials, and the spin Seebeck effect is one of the most interesting phenomena [16,108,109,110,111,112].

The spin current caused by the thermal gradient has been detected by the inverse spin Hall effect [113,114,115]. Graphene, by virtue of its highly efficient spin transmission [116,117,118], energy-dependent carrier mobility, and novel density of states [3,119], has become the focus material in this direction.

(4) $$S_{Mott}=\frac{\pi^{2}k_{B}^{2}T}{3e}•{\frac{d\ln R}{d\mu }}_{|\mu =\mu_{F}}$$

where $k_{B}$ is the Boltzmann constant, *R* is the resistance and $\mu_{F}$ is Fermi energy.

Researchers prepared graphene-based detection devices and measured their properties and spin thermoelectric parameters. The results show that the thermal gradient of the carriers in a graphene-based transverse spin valve can lead to increased spin voltage in areas near the graphene charge neutrality point (CNP). Similar to the thermal voltage in a thermocouple, the effect caused by the thermoelectric spin voltage can be enhanced by the thermal carrier generated by applying the current [120,121,122,123]. These results and research methods such as maintaining the purity of the spin signals through the thermal gradient and adjusting the remote spin accumulation by changing the spin injection bias voltage are very important for driving graphene-based spin electronic devices through thermal spin.

**Figure 14:**
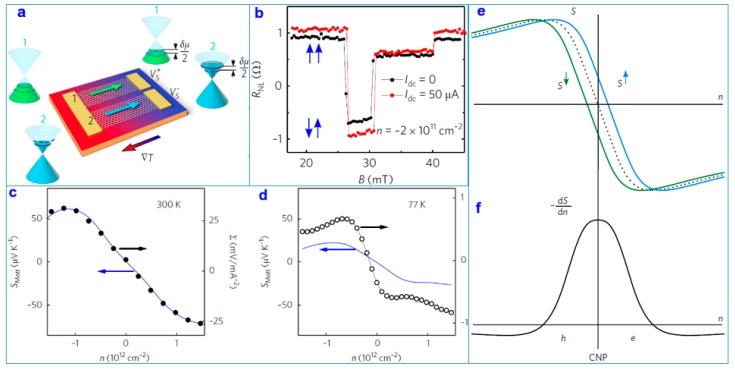
(**a**) Schematic of the test device. (**b**) Nonlocal spin resistance *R*_NL_ versus magnetic field *B* along the magnetization of the electrodes for *I*_dc_ = 0 (black line) and *I*_dc_ = 50 μA (red line). Blue arrows show the relative direction of the ferromagnetic magnetization. (**c**) Comparison between the Mott Seebeck coefficient *S*_Mott_ versus carrier density *n* (blue line and left axis) obtained from the graphene square resistance *R* at room temperature and the quadratic fitting coefficient Σ versus *n*. Modelling and roles of the Seebeck coefficient and the spin accumulation in (**e**,**f**), with qualitative representation of S (**a**) and its derivative dS/dn (**b**) about the CNP [124]. Copyright 2018, Springer Nature.

Figure 14a shows the experimental device, which consists of two graphene nanosheets (GNs) [124]. Different carrier concentrations on the GNs lead to varying Seebeck coefficients *S*_1_ and *S*_2_. The temperature difference ∆*T* on both sides of the nanosheets leads to thermoelectric voltage versus ($V_{s}=V_{s}^{+}-V_{s}^{-}=-(S_{2}-S_{1})\Delta T$), which is caused by the temperature gradient ΔT. When the carrier concentrations $n_{1}=-n_{2}$, $V_{S}=\delta \mu /e$. Figure 14b demonstrates that nonlocal spin resistance ***R***_NL_ changes slightly with the magnetic induction curve when the carrier concentration is *n* = −2 × 10^11^ cm^−1^ and the electrode current is *I*_dc_ = 0 (black line) and *I*_dc_ = 50 μA (red line), respectively. Figure 14b clearly indicates that the ferromagnetic property of the system switches (the blue arrow is the relative direction of the ferromagnetic magnetization) when the magnetic induction intensity is at *B*_1_ = 30 and *B*_2_ = 40. Figure 14c,d demonstrates the characteristics of the device. Figure 14e,f shows the qualitative representation of S (a) and its derivative *dS/dn* (b) about the CNP.

## 5. The Recent Applications in Electronic and Thermal Properties of Graphene

The novel electrical and thermal properties of graphene have been gradually recognized, and more applications are being widely used in photoelectric and thermoelectric devices.

### 5.1. Highly Efficient Thermal Conductivity Composite Film and Flexible Lateral Heat Spreaders

Polymer composite materials are ideal for horizontal heat dissipation in electronic equipment. Researchers have prepared polymer composites with highly efficient TC [125,126,127,128,129].

**Figure 15:**
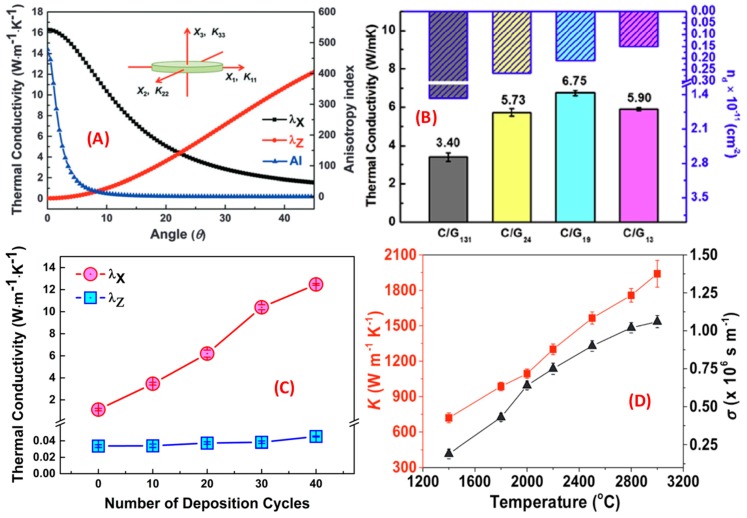
(**A**) Effect of graphene sheet orientation on theoretical heat transfer calculated by effective medium approximation (EMA) [125]. Copyright 2015, Royal Society of Chemistry. (**B**) The relationship between defects and thermal conductivity of graphene [126]. Copyright 2017, Elsevier. (**C**) The test results of thermal conductivity of a composite film [127]. Copyright 2017, American Chemical Society. (**D**) The thermal and electrical conductivity of debris-free graphene films (df-GFs) annealed at different temperatures [128]. Copyright 2018, Wiley.

Ding et al. produced a graphene–nanocellulose composite film using vacuum-assisted self-assembly (VASA). The highly crystalline nanofibers driven by natural forces are conducive to the formation of thermal conduction paths; see Figure 15a [125]. Graphene’s orientation was analyzed using effective medium approximation (EMA) to improve the TC of the composite. They changed the TC of the film by changing the defects of graphene; see Figure 15b. Through the qualitative and quantitative characterization of graphene’s defects, the increase in the defects makes the TC decrease [126]. They fabricated composite films with high in-plane TC and thermal anisotropy using layer-by-layer assembly (LBL). The results show that when the content of graphene is reduced to 1.9 wt %, the in-plane TC of the composite film reaches 12.48 Wm^−1^ K^−1^ and the anisotropy coefficient is 279; see Figure 15c [127]. Composite films have great research and application potential in various fields due to their excellent TC preparation and adjustment methods. Figure 15d shows the TC of a composite film prepared by another group of researchers [128].

### 5.2. Thermal Conductance Modulator

**Figure 16:**
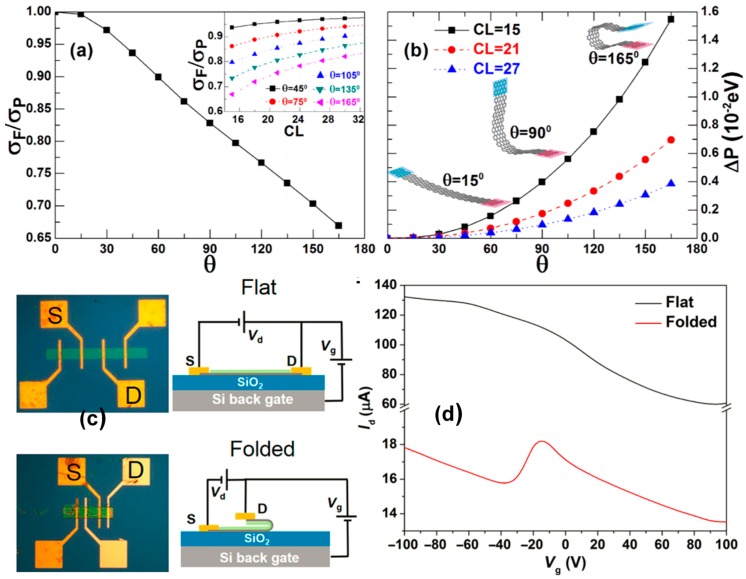
(**a**) $\sigma_{F}/\sigma_{P}$ versus *θ* for the A-FGNR-α with CL(center region length) = 15 at *T* = 300 K. The inset shows the different *θ* values belonging to $\sigma_{F}/\sigma_{P}$ versus CL [130]. Copyright 2011, American Physical Society. (**b**) The ΔP versus *θ* for the A-FGNR-α at different values of CL. (**c**) The optical images and circuit diagrams of graphene field-effect transistors (FETs); flat (top) and folded (bottom). (**d**) The transfer curves of the functionalized graphene FETs as a function of back-gate voltage in the flat (black line) and folded (red line) states [131]. Copyright 2017, Science.

Based on graphene’s robust TC and strength, a graphene nanoribbon-based TC modulator was developed in 2011 [130]. By changing the geometry of the graphene nanoribbons, the researchers were able to control and reverse the thermal conductance. This regulation can alter the conductance of unfolded graphene nanoribbons by up to 40%, as shown in Figure 16a,b. At this point, the folding angle of the GNRs exhibits a linear relationship with the conductivity and changes with the distance between the graphene layers. This thermal device has potential for use in phonon circuits and nanoscale thermal management.

Another interesting study was based on the 3D graphene structure of a curved fold that was transformed by planar graphene [131]. The distinctive properties of self-folding 3D graphene using multiscale molecular dynamics models have been revealed, making it possible to encapsulate cells or construct folded transistors; Figure 16c,d shows the results of this experiment.

### 5.3. Graphene Microheaters Based on Slow-Light-Enhanced Energy Efficiency

With high TC, graphene absorbs only 2.3% of light, which indicates that it is almost transparent. Based on these two properties, graphene is the best alternative to traditional metal thermometers. Graphene as a thermal microheater can be closely attached to the surface of an optical waveguide without considering the loss of graphene due to light absorption [132], while graphene’s high TC can quickly transfer heat to the optical waveguide, thereby enhancing the speed regulation.

**Figure 17:**
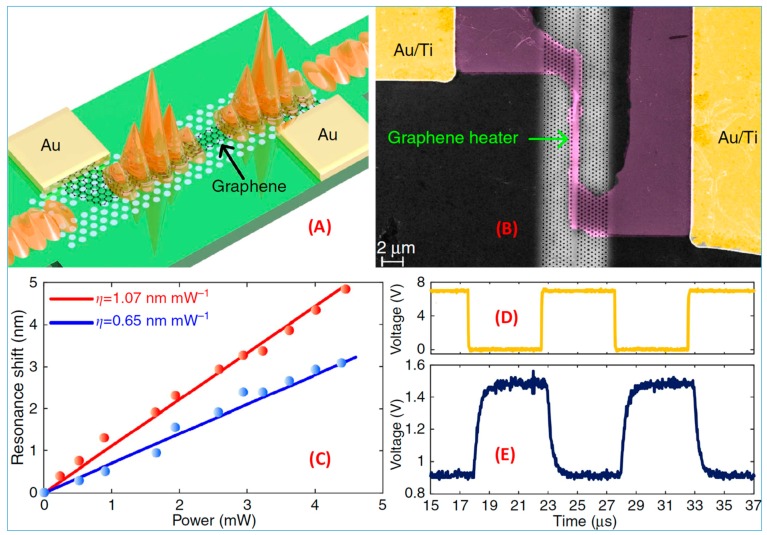
(**A**) Schematic of the slow-light-enhanced graphene heater. (**B**) False-color scanning electron microscope image of the slow-light-enhanced graphene heater. (**C**) Measured resonance shifts for the interference dips at 1525.12 nm (blue) and 1533.71 nm (red) as functions of the applied heating power. (**D**) Driving electrical signal and (**E**) corresponding temporal response signal [132]. Copyright 2017, Springer Nature.

Figure 17a,b demonstrates graphene microheaters based on slow light enhancement by placing graphene on a photonic crystal waveguide with the light propagation speed reduced to 1/30 of the vacuum. The effective heating length of the optical signal is greatly increased, thus reducing the energy loss of the optical signal. Figure 17c shows the results of a graphene thermal microheater. The thermal regulation efficiency of the device is as high as 1.07 nm·mW^−1^, which is nearly double that of traditional devices. The energy consumption of the optical signal reaching 2P phase shift is 3.99 mW, which is lower than that of traditional metal thermal heaters. The optical signal switching speed is 550 ns, which is three orders of magnitude faster than traditional metal thermal microheaters and is far from that of the fastest regulated nanothermal microheater; see Figure 17d,e. The comprehensive evaluation index of the device is 2.5413 mW, which is 30 times higher than the comprehensive evaluation index of the best nanothermal microheater. This study is expected to be widely used in integrated phased array radar systems and optical arbitrary waveform generators.

### 5.4. Hybrid Graphene Tunneling Photoconductor

Composite photodetectors formed using highly efficient optical materials (such as quantum dots, carbon nanotubes, etc.) and graphene have attracted extensive attention. The photogenerated carriers in the absorbent materials can be effectively transferred to the graphene channel with high mobility to achieve superhigh light response gain [133,134,135,136,137,138]. However, due to a large number of trap states at the interface between the absorbent materials and the graphene, this type of photodetector is usually slow in response, which restricts its use in high-frequency applications.

**Figure 18:**
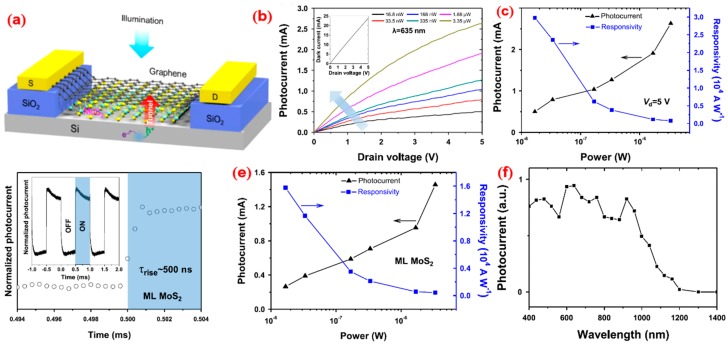
(**a**) Schematic diagram of a hybrid graphene photoconductor. (**b**) Photocurrent vs. drain voltage under various light powers at 635 nm wavelength. The arrow indicates the direction of light power increase. The inset shows the dark current of the device. (**c**) Power-dependent photocurrent and photoresponsivity at 5 V drain voltage calculated from the data in (**b**). (**d**) Normalized photocurrent vs. illumination wavelength. (**e**) Transient characteristics of the hybrid graphene photoconductor with MoS_2_ under 635 nm illumination, showing a rising time of ~500 ns. Inset is the switching performance over three periods of square-wave modulation. (**f**) Photocurrent and responsivity as functions of the illumination power of the device with MoS_2_ [139]. Copyright 2017, Springer Nature.

Based on graphene/Si hybrid photodetectors, researchers inserted single-layer MoS_2_ between graphene and silicon to improve the performance of the photoconductor [139]. The experimental results indicate that the photogenerated carriers flow out of the silicon and enter the graphene channel through the potential barrier of MoS_2_ through the quantum tunneling effect under the condition of illumination. There are no suspension bonds on the surface of the molybdenum disulfide, which reduces the trap state and effectively passivates the interface. Comparing the detection performance of devices before and after MoS_2_ insertion, the response speed of the latter is three orders of magnitude higher than the former; that is, the response time is 17 ns and the response degree is 3.4 × 10^4^ A/W. Figure 18a presents a schematic diagram of the device. Figure 18b,c,d shows the device’s photoelectric response characteristics. Figure 18e,f demonstrates the experimental results of the device. This type of nanodevice graphene-based heterostructure shows excellent performance, which provides new applications for graphene-based electrical characteristics.

### 5.5. Graphene Electrode

P-doped graphene-based electrodes improve the performance of the device by reducing its resistance. However, the resistance of the electrode will gradually increase in the environment, which will affect the actual use of the graphene electrode [140,141,142,143,144,145,146].

Researchers recently used perfluorinated polymeric sulfonic acid (PFSA) as a dopant molecule to conduct p-doped graphene to build a PFSA-based p-doped graphene electrode [146]. The electric dipole of the sulfonate group proton in the PFSA molecule strongly attracts electrons, which leads to high ionization potential of the perfluorocarbon skeleton. Doped with PFSA graphene electrodes, the device’s surface resistance (*R*_sh_) decreased by 56% and its surface potential increased by 0.8 V. Moreover, the graphene electrode of this configuration, although treated with a chemical agent, was stable under high temperatures and long-term exposure to air. This graphene-based electrode can be used to produce phosphorescent organic light-emitting diodes with high hole injection and substantial luminescence efficiency.

**Figure 19:**
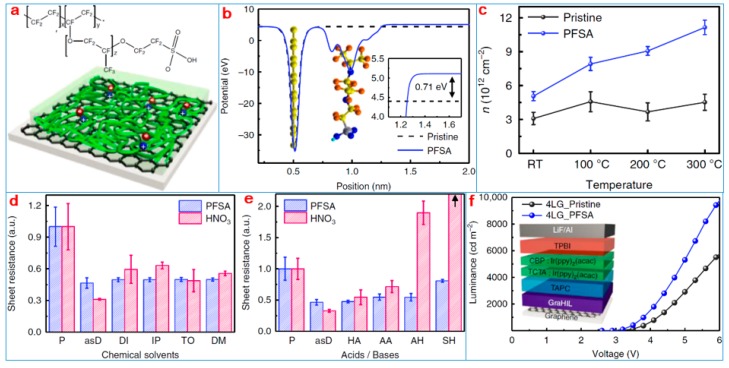
(**a**) Chemical structure of perfluorinated polymeric sulfonic acid (PFSA) and schematic drawings of graphene doped using PFSA (+: hole, −: electron). (**b**) Calculated electrostatic potential of the most stable configuration of PFSA-doped graphene (inset: difference in work function between pristine and PFSA-doped graphene). (**c**) Averaged *n* of thermally annealed pristine and PFSA-doped graphene with various *T*_a_ calculated from Raman spectroscopy results. (**d**) Various solvent treatments and (**e**) acid and base treatments as a function of exposure time. (**f**) Luminance vs. voltage of green phosphorescent organic light-emitting diodes (OLEDs) with pristine and PFSA-doped graphene anodes (inset: schematic device structure of OLEDs) [146]. Copyright 2018, Springer Nature.

Figure 19a presents a structural diagram of the PFSA-doped graphene electrode. Figure 19b shows the calculated results of the device. Figure 19c–e demonstrates the performance examination results of the electrode. Figure 19f shows the performance test results of organic light-emitting diodes (OLEDs) based on the graphene electrode. The construction of a doped graphene electrode has consistently been a popular topic of research, but many researchers focus on the regulation of its electrical properties and ignore the problem of its stability. The study used organic macromolecules as dopants to improve the electrical properties of graphene and its stability. This work can promote the construction of a stable graphene electrode and its application.

### 5.6. Dirac-Source Field-Effect Transistors (DS-FETs)

The development trend of integrated circuits has changed from the pursuit of performance and integration to the most effective way to reduce power consumption, which is to reduce the working voltage. Currently, the working voltage of the integrated circuit (14/10 nm technical node) of a complementary metal-oxide semiconductor (CMOS) is reduced to 0.7 V, while the thermal excitation limit (60 mV/decade (Dec)) of the MOS transistor’s subthreshold swing (*SS*) makes it impossible to reduce the working voltage of the integrated circuit to below 0.64 V. Existing transistors that are tunneling FET and negative capacitance FET can realize *SS* < 60 mV/Dec, but they have a low speed or important defects such as poor stability, unfavorable integration, and thus lack of practical value. The ultralow power consumption transistor to be used in future integrated circuits not only must obtain *SS* < 60 mV/Dec, ensuring the open state current is sufficiently large, but also requires stable performance and simple preparation [147,148,149,150,151,152,153,154,155,156,157].

**Figure 20:**
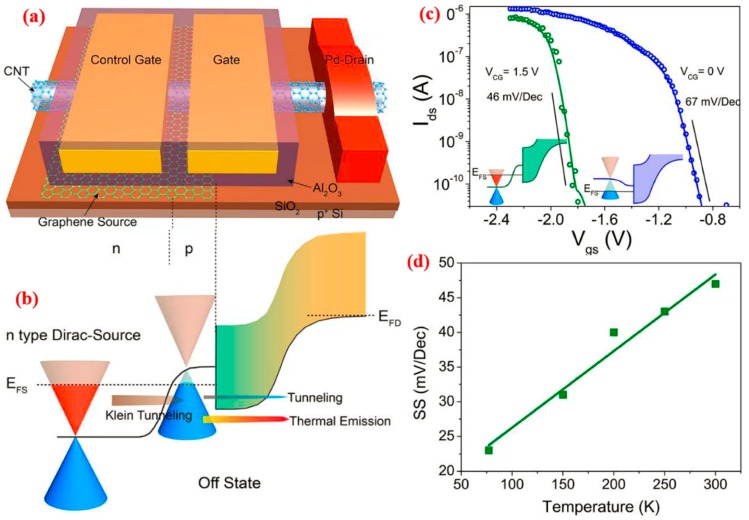
(**a**) Schematic diagram showing a Dirac-source field-effect transistor (DS-FET) with a control gate in addition to the normal gate. (**b**) Schematic diagrams illustrating the off-state of the DS-FET. (**c**) Transfer characteristics of a typical DS-FET at different *V_CG_*. Circles and lines represent experimental and simulated results, respectively. Green color represents results obtained at *V_CG_* = 1.5 V, and blue represents those obtained at *V_CG_* = 0 V. Inset figures are schematic band edge profiles for fitted data situations. (**d**) Temperature-dependent SS of a typical DS-FET measured at temperatures between 77 K and 300 K; *V_CG_* was set at 2 V to keep the device in Dirac-source mode. SS varied by more than 100% from 77 to 300 K. In all measurements, the substrate was biased with −20 V to keep the ungated region near the drain open [158]. Copyright 2018, Science.

Researchers in Beijing recently reexamined the MOS transistor and the physical limits of its threshold swing [158]. They proposed a new type of ultralow power field effect transistor and adopted doped graphene as a “cold” electronic source with carbon nanotubes as the active channel. Semiconductor sources with high-efficiency top grid structures have been built as DS-FETs. The threshold value of swing experiments has been implemented at 40 mV/Dec at room temperature (Figure 20). The results of variable temperature measurement indicate that there is an obvious linear relationship between the DS-FET subthreshold amplitude and temperature. This indicates that the carrier transport of transistors is a traditional thermal emission mechanism rather than a tunneling mechanism. The DS-FET has excellent scalability. When the channel length of the device decreases to 15 nm, it can still achieve a subthreshold swing of 60 mV/Dec.

Most importantly, the DS-FET has a proposed driving current much higher than that of tunneling transistors compared to metal-oxide semiconductor field-effect transistors, and its *SS* < 60 mV/Dec spans a larger range of currents. As the key parameter of the comprehensive index of open and closed state characteristics of sub-60 mV/Dec (that is, the current at *SS* = 60 mV/Dec), *I*_60_ = 40 μA/μm, which is 2000 times the published best tunneling transistor and fully meets the standards of the international semiconductor development roadmap (ITRS) for the practical application of sub-60 mV/Dec devices. The open and closed current of a typical DS transistor at a working voltage of 0.5 V is equivalent to that of a CMOS device at 14 nm (at a working voltage of 0.7 V). This indicates that the DS transistor can meet the requirements of future ultralow power consumption (*V*_dd_ < 0.5 V) integrated circuits. Moreover, the device structure of the DS does not rely on semiconductor materials and may be used in conventional CMOS transistors and field effect transistors in two-dimensional materials.

## 6. Conclusions and Prospects

Graphene, with its exceptional physical and chemical properties, has been increasingly applied in various fields of scientific research. When graphene’s nanostructure changes (such as in terms of boundary configuration, shape, own defects, chemical doping, and the formation of heterogeneous structures, etc.), its physical and chemical properties show novel properties. With the improvements in the preparation of graphene nanomaterials and the enhancement of their measurement and regulation, more graphene nanomaterials and their hybrid structures have been applied in electronic, photothermal, thermoelectric, and photoelectric fields.

However, graphene’s band gap characteristics limit its application. Graphene’s physical and chemical properties are both closely related to its electronic properties. Graphene’s electronic properties change its physical and chemical properties. There are two kinds of methods. One is a physical approach (for example, the change of the graphene nanostructures, the applied electric field or magnetic field, vertical configuration heterostructure or plane heterostructure, substrate, etc.). The other is a chemical method (chemical doping, other atoms or groups of adsorption, the use of chemical reagents, etc.). The fabrication level of graphene-based microscale or nanoscale devices also determines their application and development. Researchers are actively working in correlated fields, and more high-performance graphene-based devices will be prepared and used in the future.
